# Effects of motor imagery training on muscle strength and co-contraction for older adults

**DOI:** 10.3389/fpsyg.2025.1441377

**Published:** 2025-07-14

**Authors:** Wan X. Yao, Bernadett Mamone, Sha Ge, Mehmed B. Bayram, Jiang Wu, Bo Y. Jiang, John Q. Zhang, Guang H. Yue

**Affiliations:** ^1^Department of Kinesiology, College for Health, Community, and Policy, The University of Texas at San Antonio, San Antonio, TX, United States; ^2^Neurelis Inc., San Diego, CA, United States; ^3^College of Sports Science, Tianjin Normal University, Tianjin, China; ^4^Center for Spinal Stimulation Research, Kessler Foundation, West Orange, NJ, United States; ^5^Rutgers New Jersey Medical School, Rutgers, The State University of New Jersey, New Brunswick, NJ, United States; ^6^Integrative Health Center, trüHealthNow, Germantown, MD, United States; ^7^School of Public Health, Jilin Medical University, Jilin, China; ^8^Center for Mobility and Rehabilitation Engineering Research, Kessler Foundation, West Orange, NJ, United States

**Keywords:** motor imagery training, mental practice, muscle strength, muscle co-contraction, aging

## Abstract

**Objective:**

This pilot study aimed to evaluate the effectiveness of motor imagery training (MIT) in enhancing maximal voluntary contraction (MVC) force among healthy older adults and inducing neural adaptations measured by electromyography (EMG) signals of both agonist and antagonist muscles, as well as the co-contraction index (CCI).

**Methods:**

Conducted with a single MIT group using a within-subject design, the study involved 12 right-handed elderly participants, with nine completing the 8-week training (5 sessions/week and 30 min/session). Elbow flexion MVC force and MVC EMG for biceps brachii (agonist) and triceps brachii (antagonist) were recorded pre- and post-training.

**Results:**

Significant improvements in muscle strength were observed following the 8-week MIT, with a notable 22% increase (*p* < 0.05). Additionally, there was a significant 27% increase in EMG amplitude for the agonist muscle (*p* < 0.05), with no notable change in EMG amplitude for the antagonist muscle. Notably, this study is the first to demonstrate a significant decrease in CCI (−32%, *p* < 0.05) following MIT.

**Conclusion:**

These findings provide further evidence of MIT’s efficacy in enhancing voluntary muscle strength through neural adaptations, particularly beneficial and safer for older individuals encountering challenges with conventional strength training methods.

## Introduction

Aging is accompanied by a decline in voluntary muscle strength. While the age-related loss of muscle mass is a significant contributor to muscle weakness, another crucial factor is the central neural drive (Yue et al., 1999; Ranganathan et al., 2001; Sale and Semmler, 2005). This encompasses reduced activity in agonist muscles and heightened activity in antagonist muscles, both of which play a pivotal role in muscle weakness (Narici et al., 2008).

Growing evidence indicates that traditional muscle strength training paradigms enhance descending cortical drive and strengthen muscle output in the elderly. The increased maximal voluntary contraction (MVC) force could result from increased net excitation of motoneurones, increased activation of prime movers, more appropriate co-contraction of synergists, and increased inhibition of antagonists (Sale, 1986). In addition to an intensified neural drive to the motor neuron pools of the prime mover muscles with strength training, changes can involve both the relative activation of different motor neuron pools and the connectivity within and between pools. These adaptations can modulate the amount of antagonist co-activation and the levels of activation of the different synergist muscles.

Co-contraction, traditionally defined as the simultaneous activation of the antagonist muscle with the agonist muscles, can affect muscle force (Sale, 1986). Research indicates that training can modify the level of co-activation. For instance, resistance training has been found to result in a significant reduction in co-contraction and an increase in maximal voluntary contraction (MVC) force in both young (Carolan and Cafarelli, 1992; LaRoche et al., 2008) and elderly individuals (Simoneau et al., 2006; LaRoche et al., 2008).

Motor imagery refers to the internal simulation of actual movements without overt movements (Jeannerod and Decety, 1995). Research indicates that motor imagery is task-specific, with the same neural correlates selectively activated during the imagery of a movement as during the actual movement (Park and Li, 2011). Training with motor imagery has proven effective in enhancing muscle force in both young (Yao et al., 2013) and elderly populations (Jiang et al., 2016; Yue et al., 2024). Additionally, research indicates that motor imagery training (MIT) can lead to a bilateral transfer effect in both motor skill acquisition and muscle strength improvement (Yao et al., 2023). A most recent meta-analysis study (Liu et al., 2023) suggests that older adults may derive greater benefits from MIT in terms of muscle force gains compared to young adults. While research consistently attributes increase in muscle strength to neural adaptations in the central nervous system (CNS) (Yue et al., 1996; Yao et al., 2013; Ranganathan et al., 2004), such as heightened CNS commands following MIT, it remains uncertain whether MIT exerts a similar impact on both agonist and antagonist muscles, potentially affecting the level of co-contraction. MIT holds significant promise in rehabilitation medicine, particularly for weak patients or frail older adults who may find conventional high-intensity strength training programs challenging or unsafe (Jackson et al., 2001). Understanding the effects of MIT on co-contraction index (CCI) of antagonist and agonist muscles will provide valuable insights for practitioners, such as physical therapists, in optimizing rehabilitation interventions. Therefore, the aim of this pilot study was to evaluate the effectiveness of MIT in reducing CCI among older adults by analyzing alterations in both agonist and antagonist muscles, specifically in terms of their force production and electromyography (EMG) amplitudes subsequent to MIT.

Based on previous studies demonstrating the effectiveness of MIT in improving muscle strength through neural adaptations (Yue and Cole, 1992; Yao et al., 2013; Ranganathan et al., 2004; Dos Anjos et al., 2022), it was hypothesized that MIT would decrease CCI, a link between the CNS and muscle activities. Consequently, this alteration of CCI was expected to lead to an increase in MVC force among older adults.

## Research methods

### Subjects

Twelve right-handed elderly individuals (73.9 ± 8.0 years, ranging from 68 to 87, 10 females) participated in the study. Hand dominance was determined by the Edinburgh Handedness Inventor (Oldfield, 1971).

### Training protocol

In this within-subject design, all participants underwent motor imagery training (MIT) for 30 min per day, 5 days a week for 8 weeks. Non-dominant (left) side elbow flexion (EF) strength was emphasized during first-person perspective MI of maximal EF contractions (internal or kinesthetic imagery). In other words, in doing that he/she attempted to activate the brain in a same manner as in a physical MVC (Yao et al., 2013; Ranganathan et al., 2004). During each trial, the participants were instructed to imagine their left arm (focus on elbow flexors) pushing maximally against the force transducer that was used for the strength measurements during the pre-training tests or against a heavy object. There were 30 trials (3 sets, 10 trials in each set) in each training session; there was a 20-s rest between trials and 3-min rest between sets; and a total of 40 training sessions were performed. Each trial started and terminated by an auditory signal that was timed and recorded on a tape recorder. To ensure that MIT was performed without overt muscle movement, we monitored EMG signal from the two elbow flexor muscles, the biceps brachii and brachioradialis, which are accessible from the skin surface. On average, the EMG amplitude during the MIT was less than 2% of the MVC EMG recorded during the pre-training strength test, which further confirms that no overt muscle activity occurred during the MIT.

### Strength measurement

In the assessment of primary elbow flexor muscles for the task of elbow flexion, which include the biceps brachii (BB), the brachialis, and the brachioradialis muscles, the BB was selected as the primary agonist muscle for testing. The antagonist was the triceps brachii muscle. The maximal elbow-flexion isometric force was evaluated to determine the effect of training on this force. The elbow-flexion force was measured with the subject seated and the left arm abducted slightly (~10° shoulder abduction) so that the arm was in a sagittal plane and the elbow (flexed ~90°) resting on a padded support at about hip height. The forearm was in an intermediate position between supination and pronation so that the palm of the hand is facing inward. The wrist was placed in the wrist cuff that is attached to an O-shaped metal frame connected to the force transducer (JR3, 45E15A-U760 100 L450, Woodland, CA). In each test session, 5 trials of elbow-flexion MVC force were performed. The timing of the MVC force task was based on a verbal count during which the subject graded the contraction force from zero to maximum in about 2 s and then maintain this force for about 3 s. The subjects were verbally encouraged to maximize the force. The transducer output for the elbow-flexion force was displayed on an oscilloscope to provide feedback for the subject and recorded on the hard disk of a personal computer. The force signals were sampled at 200 Hz. All the strength measurement conditions were kept the same across all measurement sessions. For more detailed information on the procedures used to measure and calculate the MVC force data, please refer to Ranganathan et al. (2004) and Yao et al. (2013).

### MVC EMG

**Figure 1:**
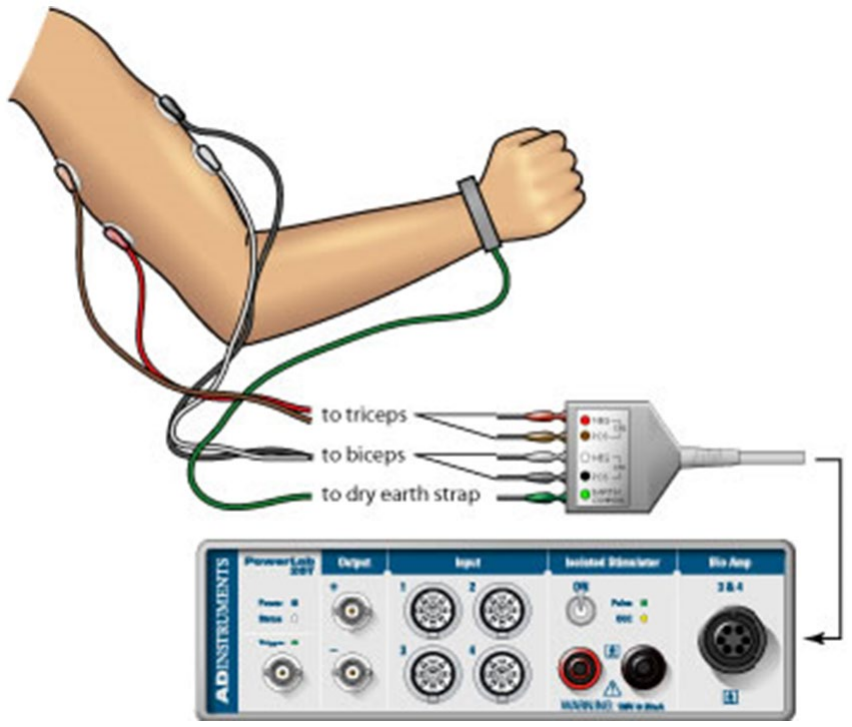
Surface EMG used to record muscle activity from the skin overlying the belly of the biceps brachii and triceps brachii muscles during the strength measurement trials.

Surface EMG was recorded from the skin overlying the belly of the biceps brachii (BB) and triceps brachii (TB) muscles during the strength measurement trials. All recordings were bipolar with the electrodes (8-mm diameter) fixed over the belly of the test muscles. All surface EMG signals were amplified (500–5,000 times), bandpass filtered (10 Hz to 1 kHz), displayed on an oscilloscope, digitized (1,000 Hz) and recorded on the computer hard disk. EMG signals were analyzed offline using the Spike2 data analysis system (Spike2, Cambridge Electronic Design, Ltd., Cambridge, UK), and custom-designed software. The signals of each trial were first full-wave rectified and then averaged across data points of 1-s duration that covered the timing of the highest force in that trial. Similar to the quantification of the MVC force, the greatest EMG amplitude among all the 5 MVC trials were taken as the MVC EMG. For more detailed information on the procedures used to measure and calculate the MVC EMG, please refer to Ranganathan et al. (2004) and Yao et al. (2013) (Figure 1).

### Co-contraction index

The CCI was determined by calculating the ratio of the EMG amplitude of the antagonist muscle to the sum of the EMG amplitudes of both the agonist and antagonist muscles during maximal voluntary contraction (MVC):


$$\mathrm{CCI}={\mathrm{EMG}}_{\text{antagonist}}/({\mathrm{EMG}}_{\text{agonist}}+{\mathrm{EMG}}_{\text{antagonist}})\times 100\%$$

### Statistical analysis

Three of the 12 participants did not complete the eight-week’s training. Thus, the analysis of MVC force and EMG amplitude were conducted using the data from the nine participants (7 females) who successfully completed the training (75.6 ± 8.5 years).

Previous studies consistently demonstrate the beneficial effects of MIT on enhancing muscle strength (Paravlic et al., 2018; Liu et al., 2023) and improving EMG amplitude on agonist muscles (Yue and Cole, 1992; Yao et al., 2013; Ranganathan et al., 2004). Therefore, the data of the MVC force, EMG amplitude, and CCI before and after the MIT were analyzed with paired-samples one-tailed *t*-tests in SPSS and the significant level was set at *p* ≤ 0.05. Given the small sample size, individual *t*-tests were chosen to minimize the risk of Type II errors and maximize the ability to detect potential meaningful changes in the outcomes.

To account for multiple comparisons, the Holm-Bonferroni correction was applied to adjust the *p*-values. This ensured that any reported findings remained statistically significant even after the adjustment for multiple tests.

## Results

### MVC force

**Figure 2:**
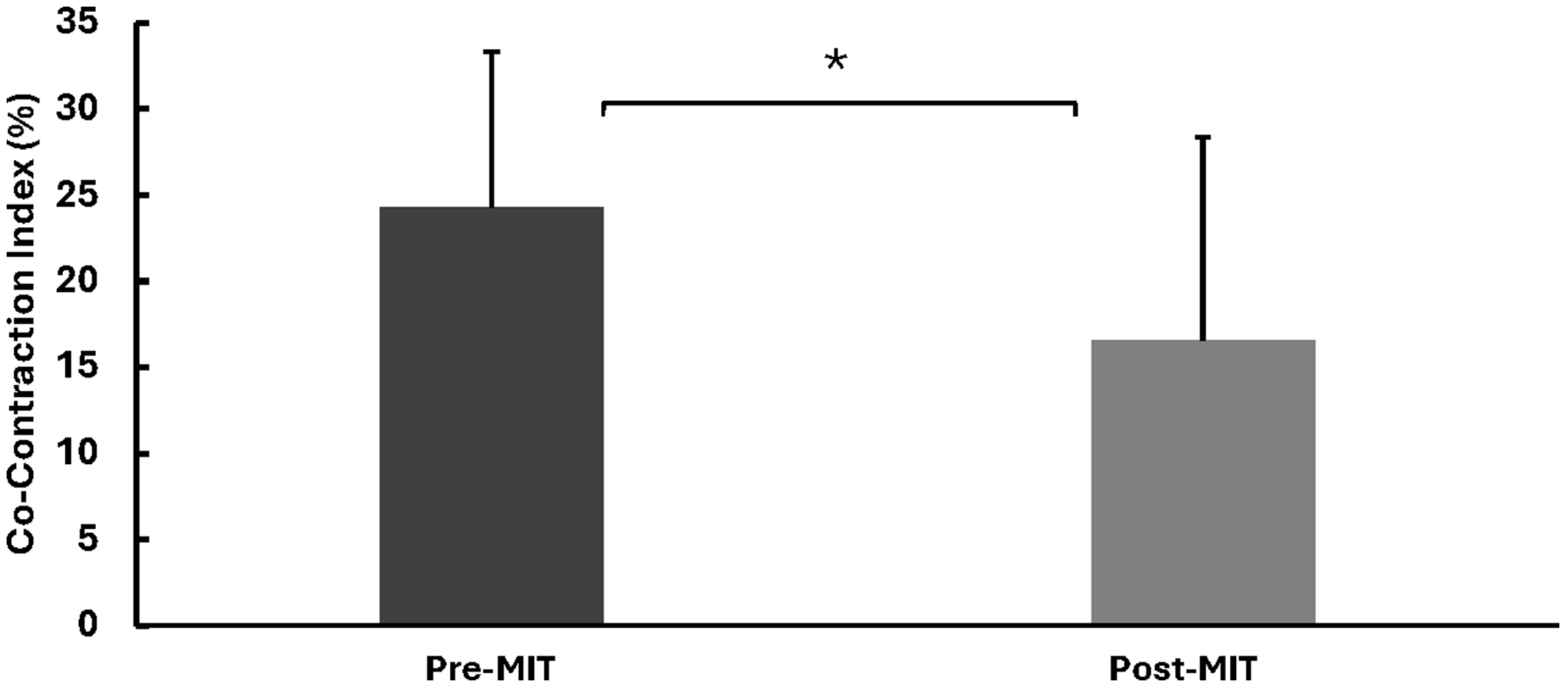
Means and standard deviations of the left EF MVC force in Newtons (N). The results reveal a significant increase of 22% (*p* < 0.05) in MVC force following the MIT. *Statistically significant at *p* < 0.05.

Following the training, there was a substantial increase (22%) in the elbow flexion (EF) MVC force. Specifically, the MVC force was 78.5 ± 38.5 N before the training, and it rose to 95.3 ± 31.3 N after the training. This difference was statistically significant, as indicated by *t*_(8)_ = 2.09, *p* < 0.05. See Figure 2 for MVC forces.

### EMG (mV) of agonist and antagonist muscles

Along with the augmented MVC force, there was a significant increase in the MVC EMG amplitude of the agonist muscle (biceps brachii, BB) following MIT. The MVC EMG a for BB were 0.51 ± 0.28 mV before the training and 0.65 ± 0.26 mV after the training, with *t*_(8)_ = 2.589, *p* < 0.05.

**Figure 3:**
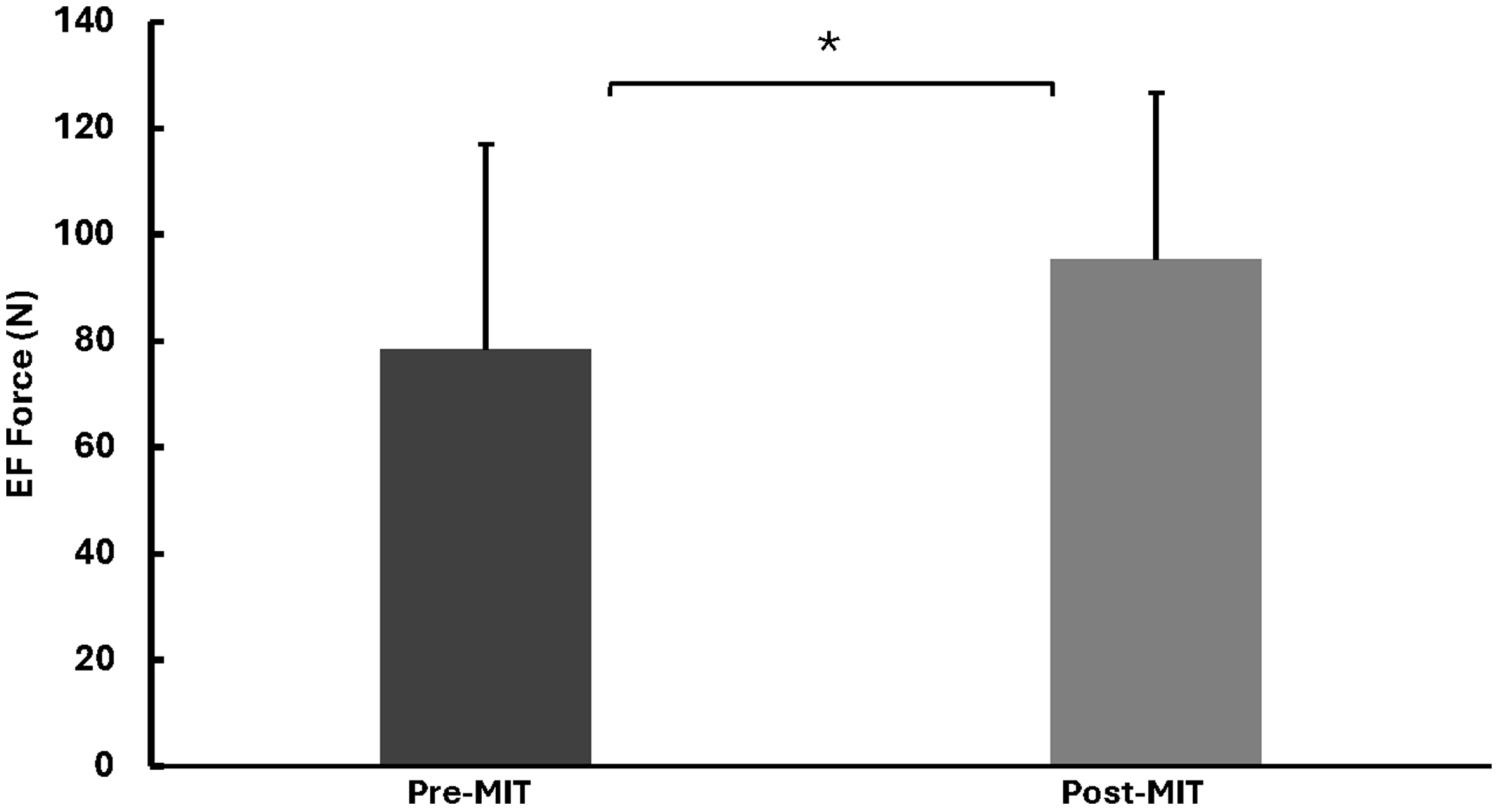
Means and standard deviations of the left BB and TB EMG amplitudes in millivolts (mV). The findings show a notable 27% (*p* < 0.05) increase for BB in EMG amplitude after the MIT and no notable difference in EMG amplitude for TB after the MIT. *Statistically significant at *p* < 0.05.

The EMG amplitude of the antagonist muscle (triceps brachii, TB) showed no significant difference before and after MIT. The mean and standard deviation of the MVC EMG amplitudes for TB were 0.11 ± 0.055 and 0.10 ± 0.065, respectively, with *t*_(8)_ = 0.275, *p* = 0.395. The EMG results are summarized in Figure 3.

### Co-contraction index

**Figure 4:**
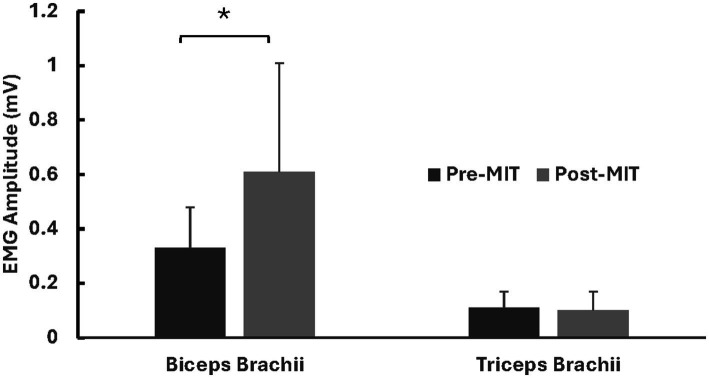
Means and standard deviations of co-contraction index (CCI). The findings reveal a significant −32% (*p* < 0.05) decrease in CCI after the MIT. *Statistically significant at *p* < 0.05.

Following the MIT, there was a significant decrease (−32%) in the CCI. The CCI values were 24.3 ± 9.01% before the training and 16.5 ± 11.7% after the training, with *t*_(8)_ = 2.028, *p* < 0.05. See Figure 4 for CCI results.

## Discussion

This pilot study investigated the effects of Motor Imagery Training (MIT) on muscle strength among older adults and explored the underlying mechanisms through analyzing the electromyography (EMG) signals. Co-contraction index (CCI) of agonist and antagonist muscles has been shown to be an important factor to the alteration of muscle strength decades ago (Carolan and Cafarelli, 1992). Therefore, in addition to assessing isometric maximal-voluntary-contraction (MVC) elbow-flexion force and EMG amplitudes of the biceps brachii (the primary agonist muscle) and triceps brachii (the antagonist muscle) during MVC, this study also examined the CCI following MIT. To the best of our knowledge, this is the first investigation to examine the effect of MIT on CCI. Our findings revealed a significant increase in muscle strength following MIT, accompanied by a noteworthy reduction in the CCI. However, the observed changes in EMG amplitude between agonist and antagonist muscles post-MIT raise intriguing questions and warrant further discussion.

This study is notable for being the first to demonstrate a significant decrease in CCI by 32% (together perhaps with strengthened descending drive to agonist muscle) following MIT, leading to a marked enhancement in muscle strength (22%). This finding aligns with previous research suggesting that motor imagery can facilitate motor unit recruitment and/or discharge rate (Yue et al., 1996; Parsowith et al., 2023; Dos Anjos et al., 2022) and induce alteration of CCI (Dos Anjos et al., 2022), thereby promoting strength gains. The effectiveness of MIT in improving muscle strength in older adults underscores its potential as a valuable intervention for enhancing physical function and mitigating age-related declines in muscle mass and strength.

Interestingly, despite the significant increase in muscle strength, our analysis revealed no concurrent change in EMG amplitude of antagonist muscles following MIT. This outcome deviates from expectations based on studies employing traditional resistance training modalities, where a decrease in EMG amplitude of antagonist muscles often accompanies increases in agonist muscle activity (Carolan and Cafarelli, 1992). This discrepancy prompts consideration of potential underlying mechanisms and implications for motor control and neuromuscular adaptations.

One possible explanation for the differential effects on agonist and antagonist muscles post-MIT could relate to the nature of motor imagery itself. Unlike physical resistance training, which directly stimulates muscle contraction through external resistance, motor imagery primarily engages neural pathways involved in motor planning and execution. It is plausible that the neural adaptations induced by MIT prioritize enhancements in agonist muscle activation, while the influence on antagonist muscles may be less pronounced or indirect. Nevertheless, compared to an increased agonist EMG, the unchanged antagonist muscle activity resulted in a relative decline in antagonist muscle EMG, leading to a reduction in the CCI.

Moreover, the specific characteristics of the motor tasks employed during MIT may influence the observed EMG amplitudes. Variations in task complexity, movement dynamics, and attentional focus during motor imagery sessions could modulate the recruitment patterns of agonist and antagonist muscles differently. Further investigations into the nuances of motor imagery protocols, including task specificity and individual variability, may provide insights into optimizing its effectiveness for improving muscle strength and coordination in older adults.

Additionally, the absence of significant changes in EMG amplitude of antagonist muscles following MIT raises intriguing questions regarding the role of reciprocal inhibition and co-contraction in neuromuscular control. While traditional resistance training often aims to minimize co-contraction and optimize agonist–antagonist muscle balance (Carolan and Cafarelli, 1992), the observed maintenance of antagonist muscle activity post-MIT suggests a more nuanced interplay between neural mechanisms governing muscle coordination in the context of motor imagery.

It should be noted that a significant limitation of the current study is the absence of a control group, which restricts the interpretability of the results. Without a control comparison, it is difficult to definitively attribute the observed changes in muscle strength and CCI specifically to MIT rather than to other factors such as learning effects, increased familiarity with testing procedures, or the Hawthorne effect. To address this limitation, we compared our findings with controlled studies of similar interventions, such as those by Yao et al. (2013) and Ranganathan et al. (2004), which demonstrated that MIT led to significant strength gains compared to control groups. The pattern of improvements observed in our study, particularly the enhancement of muscle strength and the increase in agonist muscle’s EMG amplitude, aligns with the neural adaptations reported in these controlled studies. While these comparisons cannot fully compensate for the lack of a control group, they provide contextual support for the hypothesis that MIT was the primary driver of the observed changes.

Additionally, the study had a gender imbalance, with only 2 males among the 9 participants, which may limit the generalizability of the findings, particularly given potential differences in neuromuscular responses between males and females. Another concern is the use of multiple t-tests instead of a multivariate approach, such as MANOVA, which would have been more appropriate for analyzing multiple dependent variables. However, given the small sample size of this pilot study, a MANOVA may have been underpowered, increasing the risk of Type II errors. Therefore, individual t-tests were chosen to assess the effects of MIT while minimizing the likelihood of overlooking potentially meaningful changes.

In support of transparent and reproducible cognitive neuroscience research, we also recognize the growing need for standardized, open-access tools in data collection and analysis. Toolboxes such as the Brain Electrophysiological Recording & Stimulation (BEST) toolbox (Hassan et al., 2022) exemplify best practices for collecting, analyzing, and sharing neurophysiological datasets. Future studies, particularly those involving EEG monitoring during MIT, would benefit from integrating such toolkits to promote consistency, reproducibility, and data sharing within the field.

Despite these limitations, the study provides promising preliminary evidence that MIT can enhance muscle strength in older adults by facilitating neural adaptations. To strengthen the evidence base, future research should include control groups, ensure balanced gender representation, adopt multivariate statistical approaches with adequately powered sample sizes, assess MI ability, and monitor engagement during MIT using EEG. Furthermore, investigating the long-term effects of MIT on motor performance, CCI, and other neural factors in older adults would offer a deeper understanding of its potential physiological and functional benefits.

## Conclusion

In conclusion, this pilot study provides preliminary evidence that MIT may enhance muscle strength and alter neuromuscular activation patterns, as indicated by the reduction in the CCI, in older adults. The observed improvements suggest that MIT could serve as a promising, non-invasive intervention to counteract age-related declines in muscle function. However, the study’s limitations—including the absence of a control group, gender imbalance, reliance on multiple t-tests, and lack of assessment of motor imagery (MI) ability and engagement—restrict the generalizability and interpretability of the results. These limitations highlight the need for caution in interpreting the findings, which should be viewed as preliminary evidence supporting the potential efficacy of MIT rather than definitive proof. To strengthen the evidence base, future research should incorporate controlled designs to account for potential confounding factors.

## Data Availability

The raw data supporting the conclusions of this article will be made available by the authors, without undue reservation.
